# Hyperactive Response of Direct Pathway Striatal Projection Neurons to L-dopa and D1 Agonism in Freely Moving Parkinsonian Mice

**DOI:** 10.3389/fncir.2018.00057

**Published:** 2018-07-30

**Authors:** Ben Sagot, Li Li, Fu-Ming Zhou

**Affiliations:** Department of Pharmacology, College of Medicine, The University of Tennessee Health Science Center, Memphis, TN, United States

**Keywords:** antidromic stimulation collision, basal ganglia, dopamine receptor, L-3, 4-dihydroxyphenylalanine (L-dopa), medium spiny neuron, striatum, substantia nigra, tetrode spike recording

## Abstract

Dopamine (DA) profoundly stimulates motor function as demonstrated by the hypokinetic motor symptoms in Parkinson's disease (PD) and by the hyperkinetic motor side effects during dopaminergic treatment of PD. Dopamine (DA) receptor-bypassing, optogenetics- and chemogenetics-induced spike firing of striatal DA D1 receptor (D1R)-expressing, direct pathway medium spiny neurons (dSPNs or dMSNs) promotes movements. However, the endogenous D1R-mediated effects, let alone those of DA replacement, on dSPN spike activity in freely-moving animals is not established. Here we show that using transcription factor Pitx3 null mutant (Pitx3Null) mice as a model for severe and consistent DA denervation in the dorsal striatum in Parkinson's disease, antidromically identified striatonigral neurons (D1R-expressing dSPNs) had a lower baseline spike firing rate than that in DA-intact normal mice, and these neurons increased their spike firing more strongly in Pitx3Null mice than in WT mice in response to injection of L-dopa or the D1R agonist, SKF81297; the increase in spike firing temporally coincided with the motor-stimulating effects of L-dopa and SKF81297. Taken together, these results provide the first evidence from freely moving animals that in parkinsonian striatum, identified behavior-promoting dSPNs become hyperactive upon the administration of L-dopa or a D1 agonist, likely contributing to the profound dopaminergic motor stimulation in parkinsonian animals and PD patients.

## Introduction

**Figure 1:**
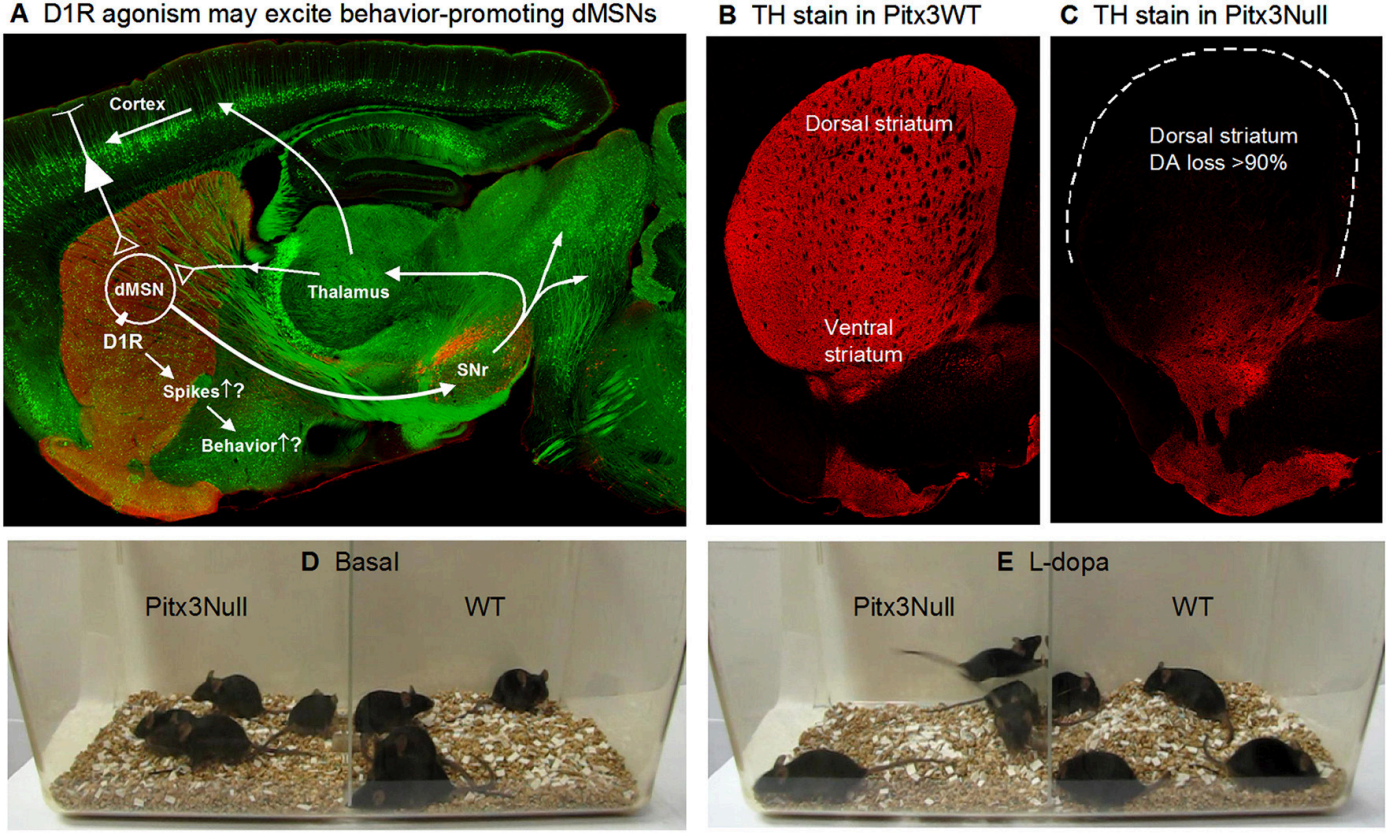
Severe DA denervation in the dorsal striatum in Pitx3Null mice. **(A)** Diagram showing the dMSN-based BG motor control circuit and the possibility that D1Rs increase dMSN spike firing. The background image shows a confocal sagittal brain section outlining the key brain structures. The red is TH stain and the green is GFP to outline the basal ganglia. **(B)** A 3 μm confocal section showing the intense DA innervation in the striatum in Pitx3WT mice. **(C)** A 3 μm confocal section showing the dorso-ventral gradient DA denervation in the striatum in Pitx3Null mice; note the severe DA denervation in the dorsal striatum.

The motor- and behavior-promoting DA system is highly concentrated in the striatum: the striatum receives an extremely dense DA innervation originated in the midbrain DA areas (Figure 1A), and the main neuronal population in the striatum, the medium spiny neurons (MSNs; also referred to as SPNs since they are the projection neurons of the striatum), express extremely high levels of D1Rs in dMSNs and D2Rs in iMSNs (Gerfen and Bolam, 2017; Zhou, 2017), providing an anatomical and molecular substrate for intense DA signaling in the striatum. Indeed, dopamine (DA) profoundly stimulates movements as demonstrated by the fact that in both animals and humans, inhibition of DA release or synthesis, or toxin destruction of the nigrostriatal DA projection, or blockade of striatal DA receptors each leads to immediate loss of motor function that is quickly restored by replenishment of DA in the striatum (Ungerstedt, 1971; Ballard et al., 1985; Zhou and Palmiter, 1995; Kim et al., 2000; Carlsson, 2001; Hornykiewicz, 2001; Galati et al., 2009; Franco and Turner, 2012; Li and Zhou, 2013; Hernández et al., 2017; Langston, 2017). Further supporting DA's motor-stimulating function, L-dopa (converted to DA once inside the brain) is the most effective clinical treatment for the motor symptoms of PD and strongly stimulates or even over-stimulate motor activity in PD patients (Katzenschlager et al., 2008; LeWitt and Fahn, 2016).

Evidence indicates that striatonigral neurons and the D1Rs intensely expressed in these neurons are critical to DA's motor function. First, DA receptor-bypassing optogenetic or chemogenetic dSPN activation stimulates motor activity, whereas inhibition or ablation of dSPNs inhibits motor activity (Kravitz et al., 2010, 2012; Durieux et al., 2012; Cui et al, 2013; Sano et al., 2013; Friend and Kravitz, 2014; Jin et al., 2014; Alcacer et al., 2017; Hernández et al., 2017; Perez et al., 2017) Second, D1R expression in dMSNs is far higher than any other neuron type in the brain (Levey et al., 1993; Yung et al., 1995; Hurd et al., 2001). Third, systemic administration of D1R agonists strongly stimulates motor activity in rodent and non-human primate PD models and PD patients (Mailman et al., 2001; Li and Zhou, 2013); equally important, microinjection of a D1 agonist into the dorsal striatum but not GPe, SNr or the motor cortex induces strong motor activity in parkinsonian animals (Wang and Zhou, 2017). Taken together, these literature data indicate a critical importance of striatal D1Rs–likely those in dSPNs–in dopaminergic motor stimulation in PD.

The question is: How does D1R and D2R agonism, the natural endogenous stimulation, affect the motor-promoting dSPN spiking activity? Despite the intense DA innervation and D1R expression in the striatum, this long-standing question had not been answered. SPN spike recordings in freely behaving parkinsonian animals are required to address these questions. However, such recordings are rare in primates (Liang et al., 2008; Singh et al., 2015, 2016) and rare and have produced conflicting results in rodents, probably due to sampling mixed dSPNs and iSPNs that may respond to DA stimulation in opposite directions (Kish et al., 1999; Chen et al., 2001; Parker et al., 2018). Thus, testing dopaminergic drugs on the spike firing in identified dSPNs and iSPNs in behaving parkinsonian animals is required, but currently data are scarce. Delineation of D1 and D2 agonistic regulation of the spike firing of dSPNs and iSPNs is necessary to advance our understanding of how L-dopa/DA affects SPN activity that in turn likely underlies DA's profound behavioral effects in PD. Information about these fundamental mechanisms will advance our understanding of basal ganglia physiology and PD pathophysiology and treatment. Thus, the goal of this study is to determine D1 agonism's effects on the spike firing of identified striatonigral neurons in freely moving DA-denervated or parkinsonian mice.

## Methods

### Animals

Animal use and the procedures were approved by the Institutional Animal Care and Use Committee of the University of Tennessee Health Science Center (UTHSC IACUC protocol # 13-056.0). This study used wild-type C57BL/6J mice and Pitx3 transcription factor-lacking C57BL/6J mice, referred to as Pitx3Null mice. Loss of Pitx3 transcription factor ultimately leads to the death of the vast majority of nigral DA neurons (Hwang et al., 2005; Zhou et al., 2016), while a subpopulation of DA neurons in the ventral tegmental area (VTA) are independent of Pitx3 and thus survive (Hwang et al., 2005; Zhou et al., 2016). Consequently, as illustrated in Figures 1B,**C**, the DA loss in the dorsal striatum is severe with > 90% of DA axons lost in the top area of the dorsal striatum, while the ventral striatum retains ~50% of DA axons originated from VTA. The residual DA is apparently sufficient to enable the mouse to retain overtly normal motor function (Figure 1D, Video 1), survive and reproduce without the need of any human intervention. The dorso-ventral gradient DA denervation resembles the DA denervation pattern in PD (Kish et al., 1988; Hornykiewicz, 2001; Kordower et al., 2013). It is amply clear that Pitx3Null mice are useful to study the consequences of DA denervation (Zhou et al., 2016). Additionally, the severe DA loss in the dorsal striatum leads to DA receptor hyperfunction in Pitx3Null mice just like that in other animal PD models and PD patients (Schwarting and Huston, 1996; Corvol et al, 2004; Wei et al., 2013, 2017; Ding et al., 2015; Li et al., 2015).

We need to note that the DA denervation in Pitx3Null mice is bilateral and likely identical among individual Pitx3Null mice, based on studies using neurochemical, immunohistochemistry and stereology (Nunes et al., 2003; van den Munckhof et al., 2003, 2006; Smidt et al., 2004; Hwang et al., 2005; Luk et al., 2013; Wei et al., 2013; Ding et al., 2015). Behavioral data also indicate that DA loss in Pitx3Null mice is bilaterally symmetric; otherwise, asymmetric rotational movement would be triggered upon L-dopa injection, but this is not the case–L-dopa does not trigger asymmetric rotational movement in Pitx3ull mice (Videos 1, 2). Further, we have documented that when the residual DA in one hemisphere was unilaterally lesioned, then L-dopa triggers rotational movements (Li et al., 2015). We also need to note that the fact that Pitx3Null mice retain largely intact motor function despite the severe DA denervation in the dorsal striatum indicates an adaptation of the DA system and the related circuits rather than a developmental adaptation, because mice can retain sufficient motor function with toxin-induced adult-onset DA denervation in the bilateral dorsal striatum (Li et al., 2015).

### Electrode microdrive implantation and electrophysiological recordings

**Figure 2:**
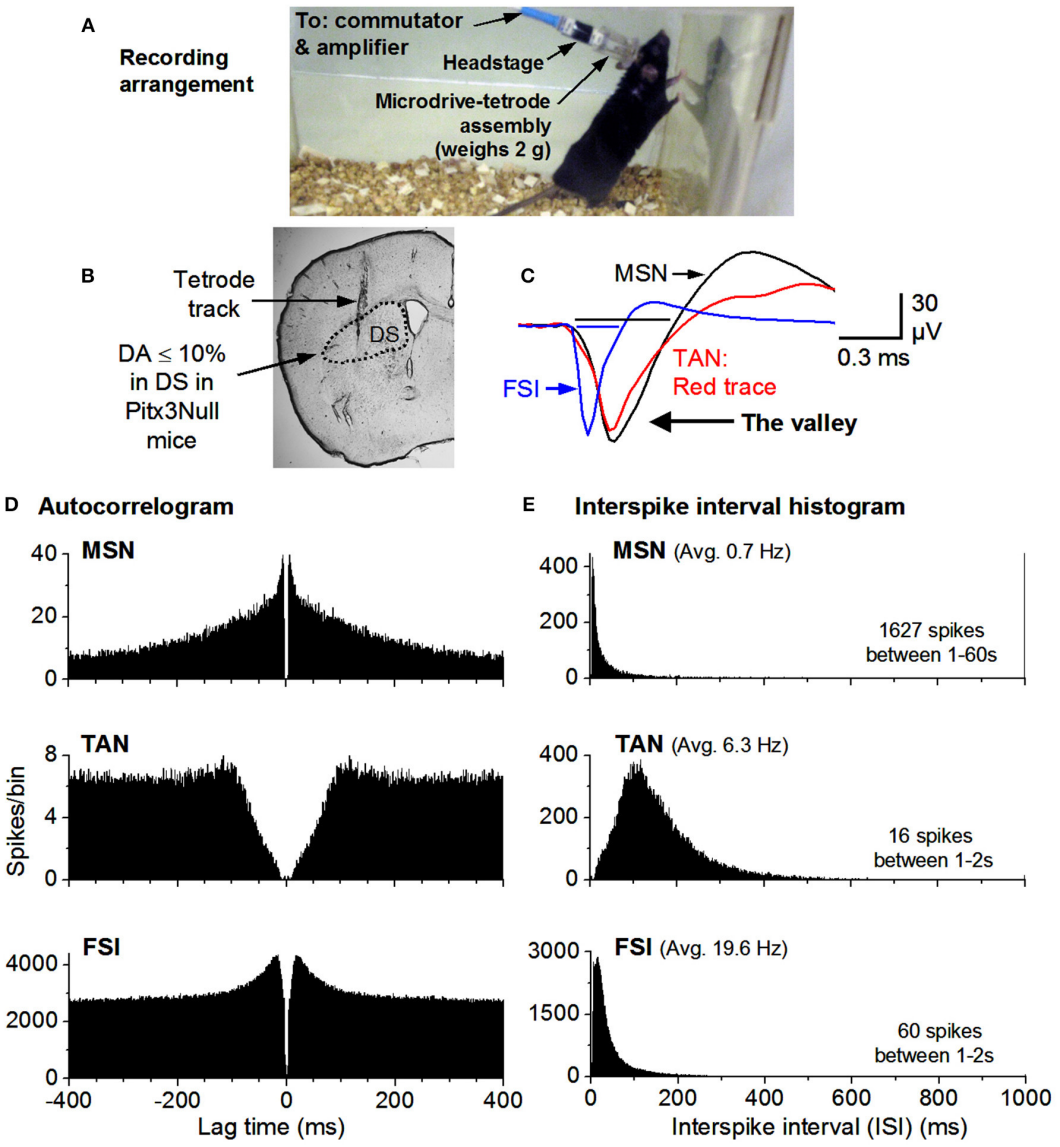
Recording arrangement and identification of MSNs, TANs, FSIs. **(A)** Recording arrangement. **(B)** A brain section showing the recording site. **(C)** FSIs have a short spike waveform (≤0.25 ms for the valley; it is ≥0.4 ms for MSNs & TANs). **(D)** Autocorrelograms show that all units have a ≥2 ms refractory period, expected for individual neurons. The TAN has a longer refractory period because TANs can not fire at high rates. **(E)** MSN firing is phasic & has a highly skewed ISI histogram. The mean ISI is listed for each example cell. The mean firing rate was 0.7 Hz for the MSN, 6.3 Hz for the TAN and 20 Hz for the FSI.

Male Pitx3Null and WT mice aged 3–6 months were used for all experiments. During recording, the animal was allowed to explore the ~8″ × 8″ × 8″ topless cage (see Figure 2). All measures were taken to eliminate or minimize the discomformt or pain the mice might feel.

The electrode microdrive arrays consisted of three or four 00–90 screws each attached to a 31 gauge stainless steel (SS) tube carrying a polytrode. Polytrodes were fabricated [per suggestions by (Liao et al., 2011); by winding (using a solution stirrer) 12 um diameter NiCr wires (Sandvik Fine Wire) into a spiraled bundle] and placed inside of a 25 ga SS tube aimed at the striatum. The entire assembly was housed inside of cylindrical plastic tubing sitting atop a polycarbonate plastic base. An electrode interface board (EIB-16, Neuralynx, Inc., Bozeman, MT) was affixed to the polycarbonate base and electrode wires were gold-pinned to the board. A non-cyanide Au solution (Gold Plating Solution, Neuralynx Inc.) was mixed with electroplating inhibitors as per Ferguson et al. (2009) and used to plate the electrode tips. (See Sagot, 2017, for more detailed procedures.)

On each animal a microdrive array containing 16 Au-plated-nichrome electrode channels in various combinations of poly-trodal configuration (bundles consisting of between 4 and 8 wires) was implanted. A stimulating electrode was included in other microdrives for stimulating antidromic spikes from the substantia nigra reticulata in order to identify striatonigral SPNs. Surgery was performed under 90 mg/kg ketamine +10 mg/kg xylazine (in 1 μL/g 0.9% saline) anesthesia following standard procedures for rodent survival surgery (e.g., Richter et al., 2013; Li et al., 2015; Wang and Zhou, 2017).

Raw recordings were pre-processed by high-pass filtering at 250 Hz with a second order elliptic filter built into the Plexon processing software (Offline Sorter, Plexon Inc., Dallas, TX) unless they had been processed in the acquisition software (Omniplex, Plexon Inc, Dallas, TX). In order to accomplish unit detection and classification the resulting pre-processed data was visualized as follows. The energy of the pre-processed data was thresholded in order to detect spike waveforms; these waveforms were initially clustered into putative single units according to contours in 3-dimensional principal component space; a template-based assignment algorithm added any remaining unsorted waveforms into said unit clusters. Published criteria utilizing auto-correlations, inter-spike interval (ISI) histograms and spike waveshape were used for classifying units to putative single SPNs, tonically active neurons (TANs), or fast spiking interneurons (FSI) (Berke, 2008; Kubota et al., 2009). High quality SPNs included in this report were characterized by ellipsoids of 1.96 standard deviations being out of contact with the nearest unit ellipsoids in a choice feature space (usually principle component space) and having <0.1% of spikes violating a refractory period of 2.0 ms. Exemplary recordings of a MSN, a TAN and a FSI are shown in Figure 2.

### Antidromic identification of SPN subtype

A parallel bipolar electrode having uninsulated tips 0.10 mm long and spaced 0.25 mm (WEST30.1A10; Microprobes, Gaithersburg, MD) was included in some microdrives for implantation into the SNr, at 3.25 mm posterior to bregma, 1.28 and 1.53 mm lateral to midline, and 4.25 mm below dura for eliciting antidromic spikes. Antidromic spikes were elicited in the axons of striatonigral neurons via varying intensities of electric current (trains consisted of either ten biphasic pulses of 0.5 ms total duration having an interstimulus interval of 100 ms or two monophasic pulses of 0.05 ms spaced 100 ms or according to potential refractory periods, i.e., 0.5–3.0 ms) applied every 20 s with a stimulus isolator (A-365; WPI, Sarasota, FL) triggered by a Master 8 (A.M.P.I., Jerusalem, Israel). These spikes were recorded in the striatum and considered as antidromically identifying if the following criteria (Ryan et al., 1989) were met: a slow latency spike response (<13 ms) time-locked to the stimulation, an all-or-none quality at peri-threshold stimulation currents, responses of progressively varying latency throughout each train corresponding to supernormal or subnormal axonal conduction velocity periods, and an absolute refractory period <2 ms (Swadlow et al., 1978; Ryan et al., 1989). Striatal neurons were considered unidentified and excluded from this report if any criterion was not met.

### Statistics

Each SPN's baseline firing rate was calculated as the mean number of spikes per second by counting all spikes recorded during the pre-drug period (defined from the saline injection +35 s through the pre-drug-injection period until 35 s before drug injection) and dividing by the duration of said period. Various metrics from every recorded SPN were calculated in and exported from MatLab (The Mathworks, Inc, Natick, MA) to an ^*^.xls file. SAS software, version 9.4 (SAS Institute Inc., Cary, NC, USA), running under Windows 7 Enterprise, was used to import this ^*^.xls file and to perform subsequent statistical analysis. Unpaired *t*-test was also used.

## Results

### Databases of mixed direct and indirect pathway SPNs

In this study, we focused on the dorsal striatum where DA denervation is severe and DA receptors are hyperfunctional in Pitx3Null mice (Wei et al., 2013, 2017; Ding et al., 2015). We recorded 46 mixed direct and indirect pathway SPNs from 7 DA-intact WT mice and 57 mixed SPNs from 7 DA-denervated Pitx3Null mice.

### Baseline dorsal striatal SPN spike firing in WT and DA-deficient Pitx3Null mice

In our datasets, the basal firing of SPNs was recorded when the mice were performing normal, natural locomotion. The basal firing rates are listed in Table 1. No significant baseline rate differences between WT and Pitx3Null mice were detected, but interpretation of this result is limited by the fact that these were mixed dSPNs and iSPNs potentially with opposite changes in basal firing rates due to DA deficiency (see Discussion).

**Table 1:** Baseline firing rates of mixed SPNs in WT and Pitx3Null mice.

	**Cell #**	**Minimum**	**Lower quartile**	**Median**	**Upper quartile**	**Maximum**
WT	46	0.00097	0.07507	0.28397	0.81032	5.94051
Pitx3	57	0.00100	0.04337	0.43982	0.52062	4.79883

### Spike responses of dorsal striatal SPNs to L-dopa in WT and DA-deficient Pitx3Null mice

Based on the published reports that D1R activation may increase dSPN excitability and D2R activation decreases iSPN excitability (Gonon, 1997; Ding and Perkel, 2002), we predicted that systemic L-dopa injection should increase the spiking activity of a population of SPNs (D1R-expressing dSPNs) and decrease the spike firing of another population of SPNs (D2R-expressing iSPNs) spike activity more strongly in Pitx3Null mice (due to hyperfunctional DA receptors) than in WT mice.

After obtaining a baseline spike recording of 30 min or more while the mouse was performing normal locomotion, 25 mg/kg L-dopa plus 10 mg/kg benserazide was IP injected. In Pitx3Null mice, within 10 min of L-dopa injection, locomotion and dyskinesia-like motor activity were stimulated, reached peaked at 20 min, plateaued for 30 min, then slowly declined over 30–45 min. In contrast, the same L-dopa injection induced no overt motor effect in WT mice. These motor behavioral observations are well established in the literature (van den Munckhof et al., 2003; Hwang et al., 2005; Li and Zhou, 2013; Wang and Zhou, 2017; Zhou, 2017).

**Figure 3:**
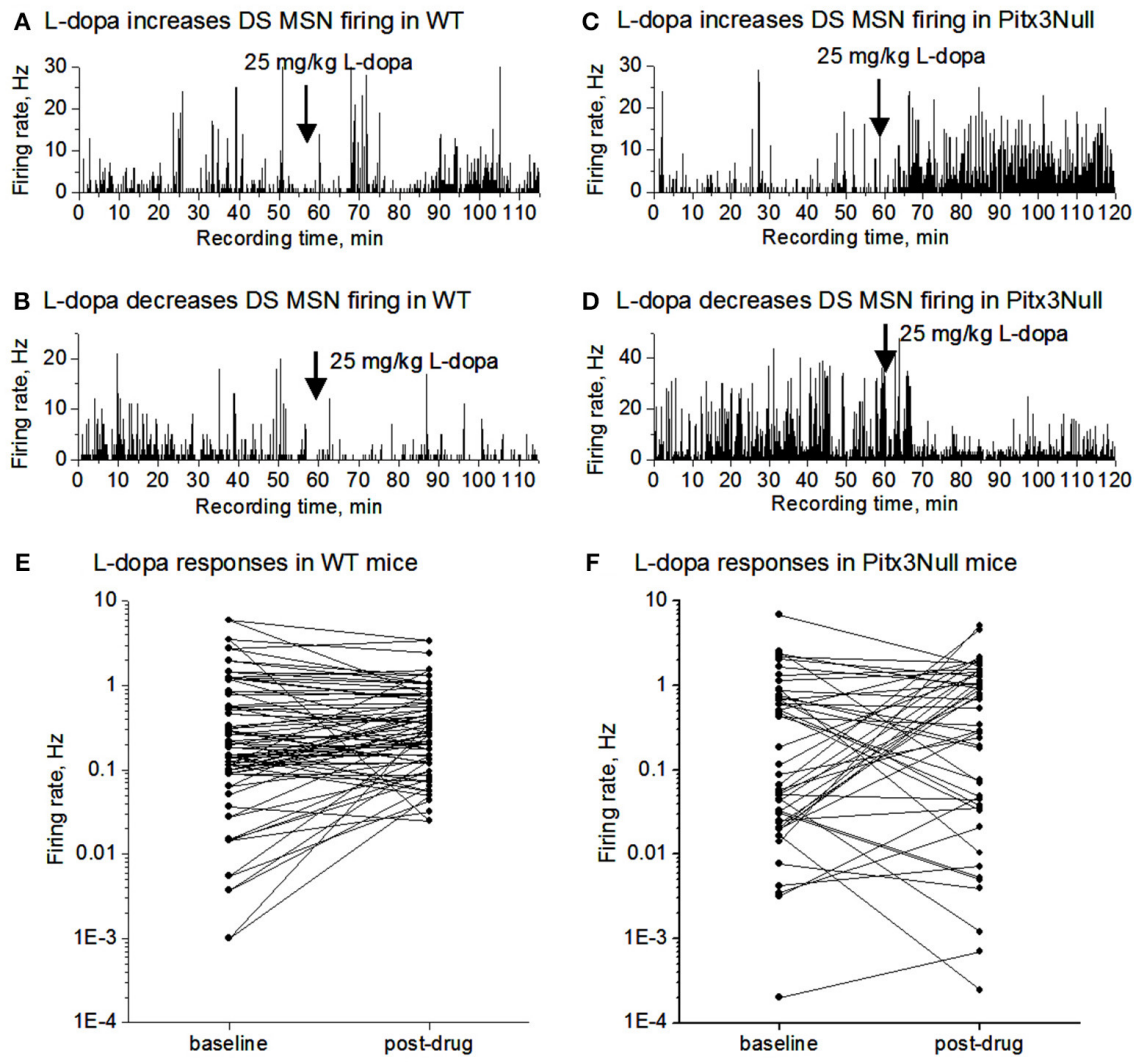
L-dopa effects on the spike firing of mixed MSNs in the dorsal striatum in DA intact WT mice and DA-denervated Pitx3Null mice. **(A)** L-dopa modestly increased the firing of a MSN in the dorsal striatum in DA-intact WT mouse. **(B)** L-dopa modestly decreased the firing of a MSN in the dorsal striatum in DA-intact WT mouse. **(C)** L-dopa strongly increased the firing of a MSN in the dorsal striatum in DA-denervated Pitx3Null mouse. **(D)** L-dopa strongly decreased the firing of a MSN in the dorsal striatum in DA-denervated Pitx3Null mouse. **(E)** Paired scatter plot of firing rates before-after L-dopa for all MSNs recorded in the dorsal striatum in Pitx3WT mice. Each dot represents the average firing rate of a single MSN. **(F)** Paired scatter plot of firing rates before-after L-dopa for all MSNs recorded in the dorsal striatum in Pitx3Null mice. Each dot represents the average firing rate of a single MSN.

We monitored SPN spike firing following IP L-dopa injection for up to 60 min in both WT and Pitx3Null mice. As shown in Figures 3A,B,E, the SPN spike response in WT mice was weak including no obvious change, a weak increase and a weak decrease among different cells. In Pitx3Null mice, SPNs also showed three spike responses: no obvious change, an increase and a decrease (Figures 3C,D,F), and the increase and decrease were apparently stronger than those in WT mice. Although the SPNs with L-dopa-induced spike firing increase may be dSPNs, and the SPNs with L-dopa-induced spike firing decrease may be iSPNs, the reliability of this conclusion is not known.

### Antidromically identified SPNs are deficient in baseline spiking and respond strongly to DA agonism

**Figure 4:**
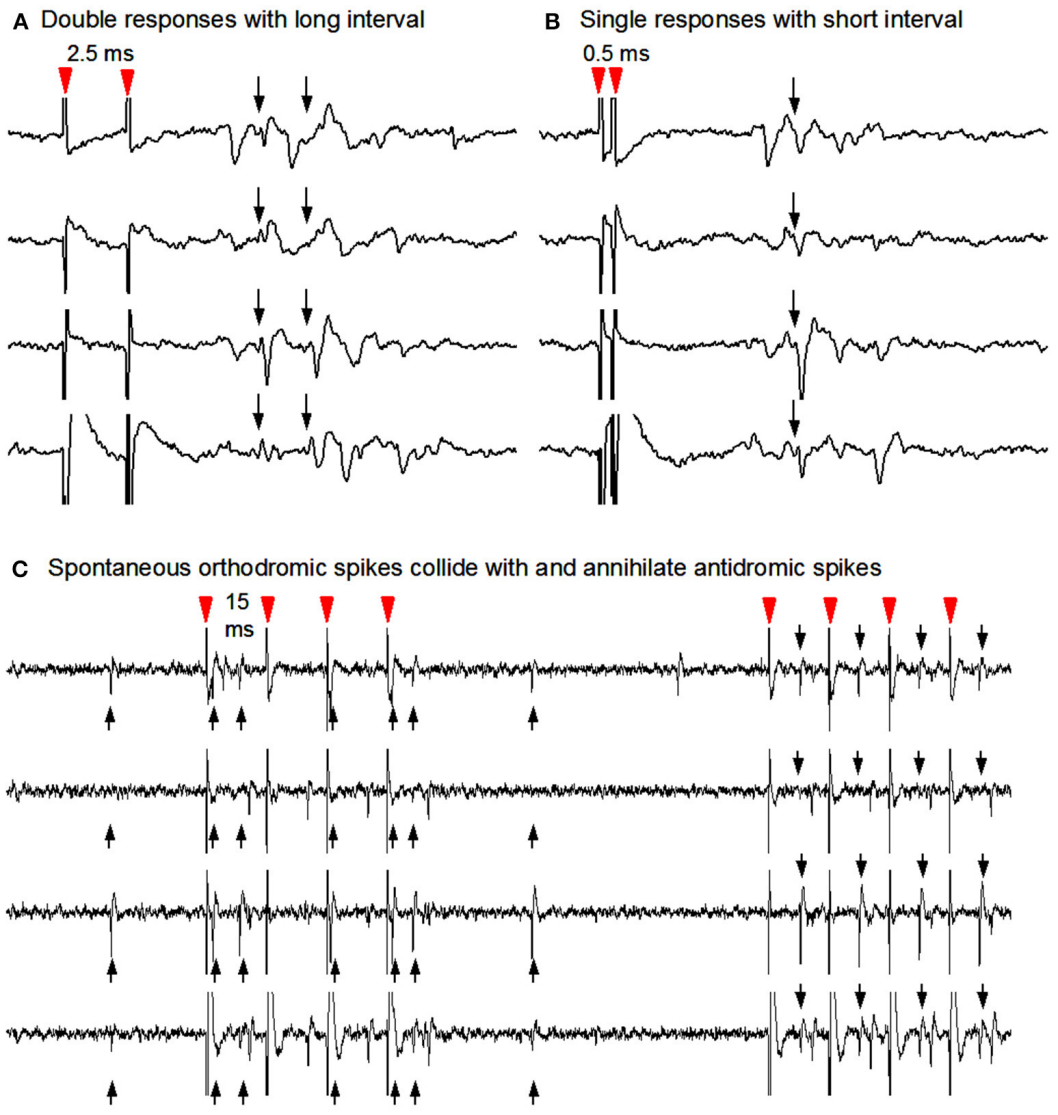
Antidromic stimulation-collision method to identify striatonigral neurons. **(A)** Nigral stimuli 2.0 ms apart elicited two corresponding spike responses at the recorded SPN soma. **(B)** Nigral stimuli using 0.5 ms interstimulus interval in order to show single response due to the Na channel inactivation-induced refactory period. **(C)** Collisions from the same unit's antidromic spikes (downward arrows) with its spontaneous orthodromic spikes (upward arrows) are evident in the first 4 stimuli and not in the latter four during which no spontaneous orthodromic spikes occurred. Note that antidromic spikes are time-locked to the stimuli (red arrow heads).

To determine the potential spike responses of identified dMSNs to dopaminergic stimulation in parkinsonian striatum, we turned to the classical antidromic activation-collision technique to identify direct pathway striatonigral (D1 receptor-expressing) SPNs (Ryan et al., 1989), taking the advantage that the striatonigral SPNs are the only SPNs projecting to the substantia nigra reticulata (SNr). As described in the Methods section and illustrated in Figure 4 here, identification was accomplished using antidromic spikes elicited by stimulating the SNr (Ryan et al., 1989). This antidromic cell identification method cannot be applied to striatopallidal neurons because large numbers of striatonigral axons go through the GPe. Thus, this study focused on striatonigral neurons.

Antidromically identified SPN baselines (all dorsal) include a total of 6 SPNs from 3 Pitx3Null animals and 8 SPNs from 3 WT animals. Non-parametric statistics for this data are shown in Table 2. The antidromically identified striatonigral neurons' baseline firing rates were found to be significantly lower in Pitx3Null mice than in WT mice.

**Table 2:** Baseline firing rates in antidromically identified striatonigral neurons.

	**Cell #**	**Minimum**	**Lower quartile**	**Median**	**Upper quartile**	**Maximum**
WT	8	0.01183	0.20093	0.31922	0.46390	5.94051
Pitx3Null	6	0.01976	0.02027	0.02640	0.08764	0.08764

**Figure 5:**
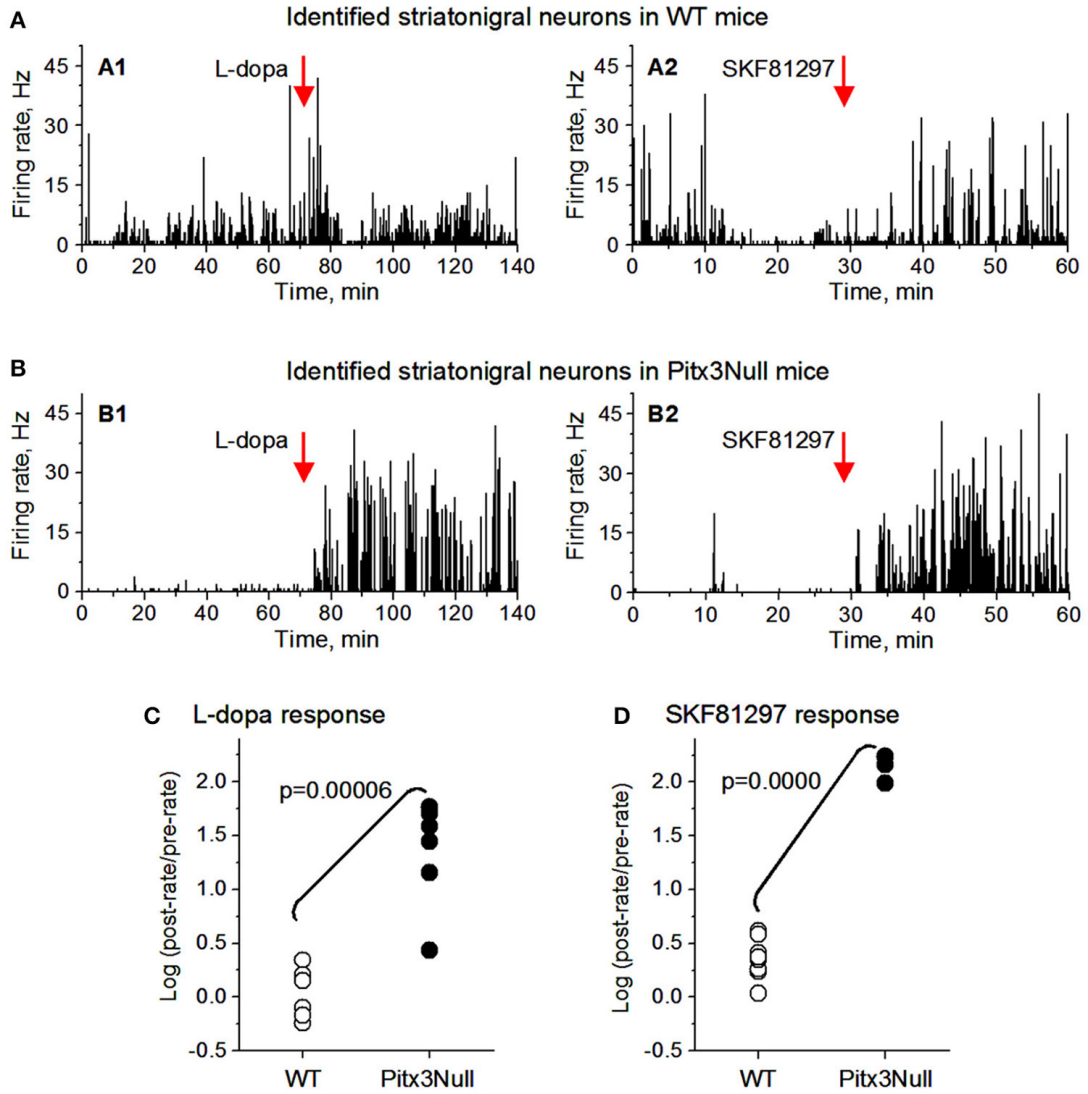
Antidromically identified dorsal striatonigral neurons responded more strongly to dopaminergic stimulation in Pitx3Null mice than in WT mice. **(A: A1,A2)**: Two examplary antidromically identified dorsal striatonigral neurons each responded weakly to L-dopa or SKF, 81297 injection in WT mice. **(B: B1,B2)**: Two examplary antidromically identified dorsal striatonigral neurons each responded strongly to L-dopa or SKF, 81297 injection in Pitx3Null mice. **(C)** Scatter plot showing that the clearly stronger responses to L-dopa in identified striatonigral neurons in Pitx3Null mice than in Pitx3WT mice. **(D)** Scatter plot showing that the stronger responses to SKF, 81297 in identified striatonigral neurons in Pitx3Null mice than in Pitx3WT mice. p values were obtained from unpaired *t*-test.

We then examined the spike responses of antidromically identified dorsal striatonigral neurons or dMSNs to IP injection of L-dopa in normal mice and DA-denervated Pitx3Null mice. Given the small sample size in the antidromically identified subset, *t*-tests were used; thus assuming SPN independence allows us to show a preliminarily significant difference between genotypes in both datasets best seen in Figure 5 as all dorsal Pitx3Null striatonigral cells had higher firing rate response intensities than WT striatonigral cells. Under these condition, we recorded and tested 6 antidromically identified striatonigral neurons from 3 WT mice. Three of these 6 neurons modestly increased and the remaining 3 striatonigral neurons modestly decreased their spiking activity in response to an IP injection of 25 mg/kg L-dopa + 10 mg/kg benserazide (Figures 5A1,C). Under the same condition, we also recorded and tested 6 antidromically identified striatonigral neurons from 3 Pitx3Null mice; and all these 6 striatonigral neurons increased their spiking activity with the increase being substantial in 5 of the 6 neurons, in response to an IP injection of 25 mg/kg L-dopa + 10 mg/kg benserazide (Figures 5B1,C). The increase in spike firing in striatonigral nigral neurons after L-dopa is clearly stronger in Pitx3Null mice than in WT mice, fully expected based on the supersensitive D1Rs in the DA-deficient Pitx3Null mice.

We next examined the spike responses of antidromically identified dorsal striatonigral neurons or dSPNs to IP injection of the D1 agonist SKF81297 in normal mice and DA-denervated Pitx3Null mice. We recorded and tested 6 antidromically identified striatonigral neurons from 2 WT mice; these striatonigral neurons modestly or moderately increased their spiking activity in response to an IP injection of 1 mg/kg SKF81297 (Figures 5A2,D). Under the same condition, we also recorded and tested 3 antidromically identified striatonigral neurons from 2 Pitx3Null mice. We found that all these 3 striatonigral neurons increased their spiking activity substantially, in response to an IP injection of 1 mg/kg SKF81297 (Figures 5B2,D). The increase in spike firing in striatonigral nigral neurons after 1 mg/kg SKF81297 injection is clearly stronger in Pitx3Null mice than in WT mice, fully expected due to hyperfunctional D1Rs in dSPNs in the DA-deficient Pitx3Null mice (Ding et al., 2015). Additionally, compared with L-dopa, SKF81297 induced a stronger increase in spike firing in identified striatonigral neurons in WT mice (Figures 5C,D), probably because SKF, 81297 can directly act on D1Rs, whereas L-dopa may not increase striatal DA release at all due to the plentiful endogenous DA in WT mice.

## Discussion

Our main findings are that the baseline spike firing of identified striatonigral neurons is lower in parkinsonian dorsal striatum than in DA-intact striatum in freely moving mice, and D1 agonism promotes spike firing more strongly in these identified striatonigral neurons in parkinsonian dorsal striatum than in DA-intact striatum in freely moving mice. These cellular abnormalities may contribute to parkinsonian motor deficits during the baseline and the supersensitive DA stimulation of motor activity in parkinsonian animals and human PD patients during dopaminergic treatment.

### L-dopa and D1 agonism each triggers hyperactive spike response in identified striatonigral neurons in parkinsonian striatum

In this study, we found that in freely moving Pitx3Null mice with DA denervation-induced DA receptor hyperfunctionality, L-dopa and D1 agonism each triggers hyperactive spike responses in identified striatonigral neurons, and this excitation temporally coincides with the motor stimulation that was characterized in our prior studies (Li and Zhou, 2013; Wang and Zhou, 2017). Although the sample size is relatively small and future confirmatory studies are needed, our data suggest that D1R agonism can induce a hyperactive response in identified dMSNs in parkinsonian striatum, providing a cellular mechanism for the hyperfunctional D1R agonistic motor stimulation (Mailman et al., 2001).

Our data are consistent with the classic basal ganglia model (Albin et al., 1989; Delong, 1990) that predicts that hyperfunctional DA receptors in the DA denervated context would allow DA agonists to lead to electrophysiological and ultimately behavioral changes via differential DA receptor activation in direct and indirect pathways. Specifically, D1 receptor signaling on dSPNs (which is coupled to the stimulatory G_alpha/olf_-cAMP cascade) (Stoof and Kebabian, 1981; Beaulieu and Gainetdinov, 2011; Hervé, 2011; Ruiz-DeDiego et al., 2015) would be activated to an unusually high degree, which would be expected to exaggerate dSPN spike firing, simultaneously supersensitive D2 receptor signaling (inhibitory Gi-coupled) would be expected to decrease the spiking of iSPNs. Together, these cellular responses would yield an overall increase in motor activity most evident in the parkinsonian condition. Thus, our present data provide some direct evidence for the classical model and its predictions on spike changes in PD and during L-dopa treatment. Our data on D1 agonistic stimulation (the endogenous physiological mechanism) of dSPNs are also consistent with the results from recent molecular, optogenetic and chemogenetic studies that clearly indicate that forced activation of dSPNs stimulates movements (Kravitz et al., 2010; Friend and Kravitz, 2014; Alcacer et al., 2017; Hernández et al., 2017; Perez et al., 2017).

We administered L-dopa and SKF, 81297 via IP injection. This could potentially complicate the interpretation of our data. However, we believe this is a minor problem, if at all, for the following reasons. First, the expression of D1Rs is concentrated in dMSNs in the brain, tens of fold higher in the striatum than in any other brain area (Levey et al., 1993; Yung et al., 1995), providing an anatomical basis for the striatum being L-dopa's primary target for motor stimulation. Second, in a comparative study conducted in our lab (Wang and Zhou, 2017), L-dopa and SKF81297 each can stimulate movements when microinjected in the dorsal striatum in Pitx3Null mice, but these drugs had weak or no motor-stimulating effect when microinjected into extrastriatal brain areas such as GPe, SNr and motor cortex, providing functional evidence that the striatal DA receptors are the primary DA receptors whose activity can profoundly stimulate movements. Third, in monkeys, drug microinjection-induced striatal DA receptor blockade is sufficient to induce PD-like motor symptoms (Franco and Turner, 2012). Thus, striatal DA receptors are the primary mediator of the profound dopaminergic motor stimulation.

The mechanisms underlying the dopaminergic enhancement of spike firing in dMSNs are currently not known. Previous studies on DA regulation of SPN excitability have produced conflicting results (Humphries and Prescott, 2010; Tritsch and Sabatini, 2012). In theory, D1R agonism may increase dSPN activity by up-regulating Na channels and down-regulating K channels and also by increasing glutamatergic synaptic inputs. These important potential mechanisms need to be determined in future studies.

We also need to note here that we have documented that in Pitx3Null mice, the L-dopa-induced motor phenotypes are due to the severity and pattern of the DA denervation, not due to the perinatal timing of DA loss; when DA loss is severe, e.g., when it reaches a 90% level, DA receptors will rapidly become supersensitive, leading to supersensitive DA behaviors, regardless of the age of the animals including humans. For example, DA receptor supersensitivity occurs within ~24 h after DA depletion in animals (Trugman and James, 1992; LaHoste and Marshall, 1994; Di Monte et al., 2000; Kim et al., 2000; Langston, 2017), and quickly in MPTP-poisoned patients (Ballard et al., 1985; Langston, 2017). Although the timing is difficult to determine, DA receptor supersensitivity certainly occurs in PD patients (Pifl et al., 1992; Corvol et al, 2004; Seeman et al., 2005) and very young children having TH deficiency (Pons et al., 2013). Furthermore, Pitx3Null mice offer the advantage that the DA loss is autogenous, PD-like gradient and consistent among individual Pitx3Null mice; thus these mice are suitable for studying the consequences of DA denervation.

Literature spike firing data from awake parkinsonian rodents are scarce, from mixed SPNs and conflicting. Chen et al. (2001) reported that apomorphine (a broad spectrum, non-selective DA agonist) modestly decreased the average firing frequency of mixed MSNs in parkinsonian striatum in awake rats, whereas Kish et al. (1999) reported that apomorphine modestly increased the average firing frequency of mixed SPNs in parkinsonian striatum in awake rats. Interpretation of these data from mixed SPNs are difficult because dSPNs and iSPNs may respond to DA stimulation in opposite directions, leading to conflicting results depending on the numbers of dSPNs and iSPNs in the mixed sample. This reasoning is consistent with Liang et al. (2008) who reported that in awake MPTP-lesioned monkeys, L-dopa excited a subgroup of SPNs that were presumed to be dSPNs and inhibited a subgroup of SPNs that were presumed to be iSPNs (Liang et al., 2008). However, some anesthetized results show an increased activity of SPNs after DA depletion in rats reportedly reduced by D1 agonists, and increased further by D2 agonists (Tseng et al., 2004; Xu et al., 2011); such data complicates interpretations arising from the canonical model. Our data–especially given such low identified cell numbers–do not exclude the possibility that D1 agonist may decrease the activity of D1R-expressing (or D2R-expressing) SPNs; further research may shed more light here and somehow expand the scope of the canonical model (e.g., Cui et al, 2013; Sippy et al., 2015). Nonetheless, our present study advances the field by recording antidromically identified striatonigral neurons and testing these neuron's responses to dopaminergic drugs in DA denervated parkinsonian mice. Our results that these striatonigral neurons become hyperactive upon DA stimulation thus may help resolve the long-standing question about what DA does to these neurons under a parkinsonian condition. Future studies need to determine the dopaminergic responses in identified iMSNs in awake parkinsonian animals and gather more identified striatonigral neurons in order to confirm our results. Since antidromic cell identification cannot be used for iSPNs because of a lack of an isolated projection from iSPNs, optogenetic and chemogenetic cell identification or other methods will be needed. Indeed, a recent Ca imaging based study indicates that L-dopa excited and inhibited dSPNs and iSPNs spike firing, respectively, in DA-depleted mice (Parker et al., 2018).

Finally, we need to discuss this question: Since Pitx3Null mice do not have overt motor deficits, are our results obtained in these mice relevant to the neuropathophysiology of the striatal SPNs in PD? We believe they are: The lack of overt motor deficits in these mice is because the severe DA denervation is restricted in the very dorsal part of the striatum, such that the motor function deficits are not expansive and hence compensated by the neighboring striatal areas; and the remaining motor deficits can not be easily detected due to the small size of mouse paws and fingers. We believe that the abnormalities in SPN activity in the DA-denervated dorsal striatum in Pitx3Null mice reported in this study likely occur in other PD models with more expansive DA denervation and PD brains, as reflected in similar molecular changes such as D1R and D2R hyperfunctionality, c-fos and pERK expression in dMSNs in PD brains and 6-OHDA DA-lesioned brains and Pitx3Null brains (Pifl et al., 1992; Corvol et al, 2004; Hwang et al., 2005; Santini et al., 2010; Ding et al., 2015). Certainly, it is possible that SPN activity abnormalities are more complex and have more network-driven components when the DA denervation area is more expansive than that in Pitx3Null mice.

## Conclusions and functional implications

By recording unit spike firing in antidromically identified striatonigral SPNs in freely moving parkinsonian mice, we have provided data supporting the hypothesis that striatonigral SPNs (i.e., dSPNs) increase their spiking activity in a hyper-responsive manner upon DA stimulation, likely contributing critically to D1 agonism's motor stimulation in parkinsonian animals. Furthermore, the baseline spike firing in these striatonigral neurons in parkinsonian striatum is lower than that in normal striatum, potentially contributing to the impaired baseline motor deficits in these mice. Finally, our data indicate that D1 agonists can activate the motor-promoting dSPNs, suggesting that D1Rs are an un-utilized treatment target for motor stimulation.

## Author contributions

BS: projection conception, data collection and analysis, manuscript writing; LL: data collection; F-MZ: projection conception, data collection and analysis, manuscript writing.

### Conflict of interest statement

The authors declare that the research was conducted in the absence of any commercial or financial relationships that could be construed as a potential conflict of interest.

## Supplementary material

Video S1 Pre-L-dopa, basal behavior of Pitx3Null mice. The 4 Pitx3Null mice (left compartment) were male, 2-month old and drug-naïve and do not show overt deficits in locomotion. The 4 Pitx3 WT mice (right compartment) were male, 2-month old and drug-naïve.

Video S2 L-dopa-stimulated behavior of Pitx3Null mice. 20 mg/kg L-dopa (together with 10 mg/kg benserazide) were IP injected 15 min ago. The Pitx3Null mice were strongly stimulated and they ran and jumped, whereas the WT did not show overt response in locomotion or other behaviors.

## References

[B1] Albin R. L. Young A. B. Penney J. B. (1989). The functional anatomy of basal ganglia disorders. Trends Neurosci. 12, 366–375. 10.1016/0166-2236(89)90074-X 2479133

[B2] Alcacer C. Andreoli L. Sebastianutto I. Jakobsson J. Fieblinger T. Cenci M. A. (2017). Chemogenetic stimulation of striatal projection neurons modulates responses to Parkinson's disease therapy. J. Clin. Invest. 127, 720–734. 10.1172/JCI90132 28112685 PMC5272195

[B3] Ballard P. A. Tetrud J. W. Langston J. W. (1985). Permanent human parkinsonism due to 1-methyl-4-phenyl-1,2,3,6-tetrahydropyridine (MPTP): seven cases. Neurology 35, 949–956. 10.1212/WNL.35.7.949 3874373

[B4] Beaulieu J. M. Gainetdinov R. R. (2011). The physiology, signaling, and pharmacology of dopamine receptors. Pharmacol Rev. 63, 182–217. 10.1124/pr.110.002642 21303898

[B5] Berke J. D. (2008). Uncoordinated firing rate changes of striatal fast-spiking interneurons during behavioral task performance. J. Neurosci. 28, 10075–10080. 10.1523/JNEUROSCI.2192-08.2008 18829965 PMC2613805

[B6] Carlsson A. (2001). A paradigm shift in brain research. Science 294, 1021–1024. 10.1126/science.1066969 11691978

[B7] Chen M. T. Morales M. Woodward D. J. Hoffer B. J. Janak P. H. (2001). *In vivo* extracellular recording of striatal neurons in the awake rat following unilateral 6-hydroxydopamine lesions. Exp. Neurol. 171, 72–83. 10.1006/exnr.2001.7730 11520122

[B8] Corvol J. C. Muriel M. P. Valjent E. Féger J. Hanoun N. Girault J. A. (2004). Persistent increase in olfactory type G-protein alpha subunit levels may underlie D1 receptor functional hypersensitivity in Parkinson disease. J. Neurosci. 24, 7007–7014. 10.1523/JNEUROSCI.0676-04.2004 15295036 PMC6729591

[B9] Cui G. Jun S. B. Jin X. Pham M. D. Vogel S. S. Lovinger D. M.. (2013) Concurrent activation of striatal direct and indirect pathways during action initiation. Nature 494, 238–242. 10.1038/nature11846 23354054 PMC4039389

[B10] Delong M. R. (1990). Primate models of movement disorders of basal ganglia origin. Trends Neurosci. 13, 281–285. 10.1016/0166-2236(90)90110-V 1695404

[B11] Di Monte D. A. McCormack A. Petzinger G. Janson A. M. Quik M. Langston W. J. (2000) Relationship among nigrostriatal denervation, parkinsonism, and dyskinesias in the MPTP primate model. Mov. Disord. 15, 459–66. 10.1002/1531-8257(200005)15:3<459::AID-MDS1006>3.0.CO;2-3 10830409

[B12] Ding L. Perkel D. J. (2002) Dopamine modulates excitability of spiny neurons in the avian basal ganglia. J. Neurosci. 22, 5210–5218. 10.1523/JNEUROSCI.22-12-05210.2002 12077216 PMC6757730

[B13] Ding S. Li L. Zhou F. M. (2015). Nigral dopamine loss induces a global upregulation of presynaptic dopamine D1 receptor facilitation of the striatonigral GABAergic output. J. Neurophysiol. 113, 1697–1711. 10.1152/jn.00752.2014 25552639 PMC4359993

[B14] Durieux P. F. Schiffmann S. N. de Kerchove d'Exaerde A. (2012) Differential regulation of motor control and response to dopaminergic drugs by D1R D2R neurons in distinct dorsal striatum subregions. EMBO J. 31, 640–653. 10.1038/emboj.2011.400 22068054 PMC3273396

[B15] Ferguson J. E. Boldt C. Redish A. D. (2009). Creating low-impedance tetrodes by electroplating with additives. Sens. Actuators A Phys. 156, 388–393. 10.1016/j.sna.2009.10.001 21379404 PMC3048465

[B16] Franco V. Turner R. S. (2012). Testing the contributions of striatal dopamine loss to the genesis of parkinsonian signs. Neurobiol. Dis. 47, 114–125. 10.1016/j.nbd.2012.03.028 22498034 PMC3358361

[B17] Friend D. M. Kravitz A. V. (2014) Working together: basal ganglia pathways in action selection. Trends Neurosci. 37, 301–303. 10.1016/j.tins.2014.04.004 24816402 PMC4041812

[B18] Galati S. Stanzione P. D'Angelo V. Fedele E. Marzetti F. Sancesario G.. (2009) The pharmacological blockade of medial forebrain bundle induces an acute pathological synchronization of the cortico-subthalamic nucleus-globus pallidus pathway. J. Physiol. 587, 4405–4423. 10.1113/jphysiol.2009.172759 19622605 PMC2766647

[B19] Gerfen C. R. Bolam J. P. (2017). The neuroanatomical organization of the basal ganglia, in Handbook of Basal Ganglia Structure and Function, eds Steiner H. Tseng K. Y. (Amsterdam: Academic Press), 3–30.

[B20] Gonon F (1997) Prolonged and extrasynaptic excitatory action of dopamine mediated by D1 receptors in the rat striatum *in vivo*. J. Neurosci. 17:5972–5978. 10.1523/JNEUROSCI.17-15-05972.1997 9221793 PMC6573191

[B21] Hervé D. (2011). Identification of a specific assembly of the g protein golf as a critical and regulated module of dopamine and adenosine-activated cAMP pathways in the striatum. Front. Neuroanat. 5:48. 10.3389/fnana.2011.00048 21886607 PMC3155884

[B22] Hernández F. L. Castela I. Ruiz-DeDiego I. Obeso J. A. Moratalla R. (2017). Striatal activation by optogenetics induces dyskinesias in the 6-hydroxydopamine rat model of Parkinson disease. Mov Disord. 32, 530–537. 10.1002/mds.26947 28256089

[B23] Hornykiewicz O. (2001). Chemical neuroanatomy of the basal ganglia—normal and in Parkinson's disease. J. Chem. Neuroanat. 22, 3–12. 11470551 10.1016/s0891-0618(01)00100-4

[B24] Humphries M. D. Prescott T. J. (2010). The ventral basal ganglia, a selection mechanism at the crossroads of space, strategy, and reward. Prog Neurobiol. 90, 385–417. 10.1016/j.pneurobio.2009.11.003 19941931

[B25] Hurd Y. L. Suzuki M. Sedvall G. C. (2001). D1 and D2 dopamine receptor mRNA expression in whole hemisphere sections of the human brain. J. Chem. Neuroanat. 22, 127–137. 10.1016/S0891-0618(01)00122-3 11470560

[B26] Hwang D. Y. Fleming S. M. Ardayfio P. Moran-Gates T. Kim H. Tarazi F. I.. (2005). 3,4-dihydroxyphenylalanine reverses the motor deficits in Pitx3-deficient aphakia mice: behavioral characterization of a novel genetic model of Parkinson's disease. J. Neurosci. 25, 2132–2137. 10.1523/JNEUROSCI.3718-04.2005 15728853 PMC6726071

[B27] Jin X. Tecuapetla F. Costa R. M. (2014). Basal ganglia subcircuits distinctively encode the parsing and concatenation of action sequences. Nat. Neurosci. 17, 423–430. 10.1038/nn.3632 24464039 PMC3955116

[B28] Katzenschlager R. Head J. Schrag A. Ben-Shlomo Y. Evans A. Lees A. J. (2008). Parkinson's Disease Research Group of the United Kingdom. Fourteen-year final report of the randomized PDRG-UK trial comparing three initial treatments in PD. Neurology 71, 474–480. 10.1212/01.wnl.0000310812.43352.66 18579806

[B29] Kim D. S. Szczypka M. S. Palmiter R. D. (2000). Dopamine-deficient mice are hypersensitive to dopamine receptor agonists. J. Neurosci. 20, 4405–4413. 10.1523/JNEUROSCI.20-12-04405.2000 10844009 PMC6772455

[B30] Kish L. J. Palmer M. R. Gerhardt G. A. (1999). Multiple single-unit recordings in the striatum of freely moving animals: effects of apomorphine and D-amphetamine in normal and unilateral 6-hydroxydopamine-lesioned rats. Brain Res. 833, 58–70. 10.1016/S0006-8993(99)01496-1 10375677

[B31] Kish S. J. Shannak K. Hornykiewicz O. (1988). Uneven pattern of dopamine loss in the striatum of patients with idiopathic Parkinson's disease. Pathophysiologic and clinical implications. N. Engl. J. Med. 318, 876–880. 10.1056/NEJM198804073181402 3352672

[B32] Kordower J. H. Olanow C. W. Dodiya H. B. Chu Y. Beach T. G. Adler C. H.. (2013). Disease duration and the integrity of the nigrostriatal system in Parkinson's disease. Brain 136, 2419–2431. 10.1093/brain/awt192 23884810 PMC3722357

[B33] Kravitz A. V. Freeze B. S. Parker P. R. Kay K. Thwin M. T. Deisseroth K.. (2010). Regulation of parkinsonian motor behaviours by optogenetic control of basal ganglia circuitry. Nature 466, 622–626. 10.1038/nature09159 20613723 PMC3552484

[B34] Kravitz A. V. Tye L. D. Kreitzer A. C. (2012). Distinct roles for direct and indirect pathway striatal neurons in reinforcement. Nat. Neurosci. 15, 816–818. 10.1038/nn.3100 22544310 PMC3410042

[B35] Kubota Y. Liu J. Hu D. DeCoteau W. E. Eden U. T. Smith A. C.. (2009). Stable encoding of task structure coexists with flexible coding of task events in sensorimotor striatum. J. Neurophysiol. 102, 2142–2160. 10.1152/jn.00522.2009 19625536 PMC2775375

[B36] LaHoste G. J. Marshall J. F. (1994). Rapid development of D1 and D2 dopamine receptor supersensitivity as indicated by striatal and pallidal Fos expression. Neurosci. Lett. 179, 153–156. 10.1016/0304-3940(94)90957-1 7845612

[B37] Langston J. W. (2017). The MPTP Story. J. Parkinsons Dis. 7, S11–S22. 10.3233/JPD-179006 28282815 PMC5345642

[B38] Levey A. I. Hersch S. M. Rye D. B. Sunahara R. K. Niznik H. B. Kitt C. A.. (1993). Localization of D1 and D2 dopamine receptors in brain with subtype-specific antibodies. Proc. Natl. Acad. Sci. U.S.A. 90, 8861–8865. 10.1073/pnas.90.19.8861 8415621 PMC47460

[B39] LeWitt P. A. Fahn S. (2016). Levodopa therapy for Parkinson disease: a look backward and forward. Neurology 86, S3–S12. 10.1212/WNL.0000000000002509 27044648

[B40] Li L. Sagot B. Zhou F. M. (2015). Similar L-dopa-stimulated motor activity in mice with adult-onset 6-hydroxydopamine-induced symmetric dopamine denervation and in transcription factor Pitx3 null mice with perinatal-onset symmetric dopamine denervation. Brain Res. 1615, 12–21. 10.1016/j.brainres.2015.04.011 25960345

[B41] Li L. Zhou F. M. (2013). Parallel dopamine D1 receptor activity dependence of L-dopa-induced normal movement and dyskinesia in mice. Neuroscience 236, 66–76. 10.1016/j.neuroscience.2012.12.065 23357114 PMC3616626

[B42] Liang L. DeLong M. R. Papa S. M. (2008). Inversion of dopamine responses in striatal medium spiny neurons and involuntary movements. J Neurosci. 28, 7537–7547. 10.1523/JNEUROSCI.1176-08.2008 18650331 PMC3343722

[B43] Liao Y. F. Tsai M. L. Yen C. T. Cheng C. H. (2011). A simple method for fabricating microwire tetrode with sufficient rigidity and integrity without a heat-fusing process. J. Neurosci. Methods 195, 211–215. 10.1016/j.jneumeth.2010.12.017 21182869

[B44] Luk K. C. Rymar V. V. van den Munckhof P. Nicolau S. Steriade C. Bifsha P.. (2013). The transcription factor Pitx3 is expressed selectively in midbrain dopaminergic neurons susceptible to neurodegenerative stress. J. Neurochem. 125, 932–943. 10.1111/jnc.12160 23331067

[B45] Mailman R. Huang X. Nichols D. E. (2001). Parkinson's disease and D1 dopamine receptors. Curr. Opin. Investig. Drugs 2, 1582–1591. 11763161

[B46] Nunes I. Tovmasian L. T. Silva R. M. Burke R. E. Goff S. P. (2003). Pitx3 is required for development of substantia nigra dopaminergic neurons. Proc. Natl. Acad. Sci. U.S.A. 100, 4245–4250. 10.1073/pnas.0230529100 12655058 PMC153078

[B47] Parker J. G. Marshall J. D. Ahanonu B. Wu Y. W. Kim T. H. Grewe B. F.. (2018). Diametric neural ensemble dynamics in parkinsonian and dyskinetic states. Nature 557, 177–182. 10.1038/s41586-018-0090-6 29720658 PMC6526726

[B48] Perez X. A. Zhang D. Bordia T. Quik M. (2017). Striatal D1 medium spiny neuron activation induces dyskinesias in parkinsonian mice. Mov. Disord. 32, 538–548. 10.1002/mds.26955 28256010 PMC5398928

[B49] Pifl C. Nanoff C. Schingnitz G. Schütz W. Hornykiewicz O. (1992). Sensitization of dopamine-stimulated adenylyl cyclase in the striatum of 1-methyl-4-phenyl-1,2,3,6-tetrahydropyridine-treated rhesus monkeys and patients with idiopathic Parkinson's disease. J. Neurochem. 58, 1997–2004. 10.1111/j.1471-4159.1992.tb10939.x 1349341

[B50] Pons R. Syrengelas D. Youroukos S. Orfanou I. Dinopoulos A. Cormand B.. (2013). Levodopa-induced dyskinesias in tyrosine hydroxylase deficiency. Mov Disord. 28, 1058–1063. 10.1002/mds.25382 23389938

[B51] Richter A. Xie Y. Schumacher A. Löffler S. Kirch R. D. Al-Hasani J.. (2013). Simple implantation method for flexible, multisite microelectrodes into rat brains. Front. Neuroeng. 6:6. 10.3389/fneng.2013.00006 23898266 PMC3721086

[B52] Ruiz-DeDiego I. Naranjo J. R. Hervé D. Moratalla R. (2015). Dopaminergic regulation of olfactory type G-protein α subunit expression in the striatum. Mov. Disord. 30, 1039–1049. 10.1002/mds.26197 25772224

[B53] Ryan L. J. Young S. J. Segal D. S. Groves P. M. (1989). Antidromically identified striatonigral projection neurons in the chronically implanted behaving rat: relations of cell firing to amphetamine-induced behaviors. Behav. Neurosci. 103, 3–14. 10.1037/0735-7044.103.1.3 2923676

[B54] Sano H. Chiken S. Hikida T. Kobayashi K. Nambu A. (2013). Signals through the striatopallidal indirect pathway stop movements by phasic excitation in the substantia nigra. J. Neurosci. 33, 7583–7594. 10.1523/JNEUROSCI.4932-12.2013 23616563 PMC6619573

[B55] Santini E. Sgambato-Faure V. Li Q. Savasta M. Dovero S. Fisone G.. (2010). Distinct changes in cAMP and extracellular signal-regulated protein kinase signalling in L-DOPA-induced dyskinesia. PLoS ONE 5:e12322. 10.1371/journal.pone.0012322 20808799 PMC2925943

[B56] Sagot B. (2017). Pitx3Null Mutant (Striatal Dopamine-Deficient) Mice Have Exaggerated Spiny Projection Neuron Responses to l-DOPA and D1 Agonism and Lack Baseline Striatonigral Spiking. Theses and Dissertations (ETD). Paper 447. 10.21007/etd.cghs.2017.0442

[B57] Schwarting R. K. Huston J. P. (1996) The unilateral 6-hydroxydopamine lesion model in behavioral brain research. Analysis of functional deficits, recovery and treatments. Prog Neurobiol. 50, 275–331. 8971983 10.1016/s0301-0082(96)00040-8

[B58a] Singh A. Liang L. Kaneoke Y. Cao X. Papa S. M. (2015). Dopamine regulates distinctively the activity patterns of striatal output neurons in advanced parkinsonian primates. J. Neurophys. 113, 1533–1544. 10.1152/jn.00910.2014 25505120 PMC4346722

[B58b] Singh A. Mewes K. Gross R. E. DeLong M. R. Obeso J. A. Papa S. M. (2016). Human striatal recordings reveal abnormal discharge of projection neurons in Parkinson's disease. Proc. Nat. Acad. Sci. 113, 9629–9634. 10.1073/pnas.1606792113 27503874 PMC5003232

[B58] Seeman P. Weinshenker D. Quirion R. Srivastava L. K. Bhardwaj S. K. Grandy D. K.. (2005). Dopamine supersensitivity correlates with D2High states, implying many paths to psychosis. Proc. Natl. Acad. Sci. U.S.A. 102, 3513–3518. 10.1073/pnas.0409766102 15716360 PMC548961

[B59] Sippy T. Lapray D. Crochet S. Petersen C. C. (2015). Cell-type-specific sensorimotor processing in striatal projection neurons during goal-directed. Behav. Neuron 88, 298–305. 10.1016/j.neuron.2015.08.039 26439527 PMC4622932

[B60] Smidt M. P. Smits S. M. Bouwmeester H. Hamers F. P. van der Linden A. J. Hellemons A. J.. (2004). Early developmental failure of substantia nigra dopamine neurons in mice lacking the homeodomain gene Pitx3. Development 131, 1145–1155. 10.1242/dev.01022 14973278

[B61] Stoof J. C. Kebabian J. W. (1981). Opposing roles for D-1 and D-2 dopamine receptors in efflux of cyclic AMP from rat neostriatum. Nature 294, 366–368. 10.1038/294366a0 6273735

[B62] Swadlow H. A. Rosene D. L. Waxman S. G. (1978). Characteristics of interhemispheric impulse conduction between prelunate gyri of the rhesus monkey. Exp. Brain Res. 33, 455–467. 10.1007/BF00235567 103739

[B63] Tritsch N. X. Sabatini B. L. (2012). Dopaminergic modulation of synaptic transmission in cortex and striatum. Neuron 76, 33–50. 10.1016/j.neuron.2012.09.023 23040805 PMC4386589

[B64] Trugman J. M. James C. L. (1992). Rapid development of dopaminergic supersensitivity in reserpine-treated rats demonstrated with 14C-2-deoxyglucose autoradiography. J. Neurosci. 12, 122875–122879. 10.1523/JNEUROSCI.12-07-02875.1992 1613560 PMC6575832

[B65] Tseng K. Y. Riquelme L. A. Murer M. G. (2004). Impact of D1-class dopamine receptor on striatal processing of cortical input in experimental parkinsonism *in vivo*. Neuroscience 123, 293–298. 10.1016/j.neuroscience.2003.10.005 14698740

[B66] Ungerstedt U. (1971). Postsynaptic supersensitivity after 6-hydroxy-dopamine induced degeneration of the nigro-striatal dopamine system. Acta Physiol. Scand Suppl. 367, 69–93. 10.1111/j.1365-201X.1971.tb11000.x 4332693

[B67] van den Munckhof P. Gilbert F. Chamberland M. Lévesque D. Drouin J. (2006). Striatal neuroadaptation and rescue of locomotor deficit by L-dopa in aphakia mice, a model of Parkinson's disease. J. Neurochem. 96, 160–170. 10.1111/j.1471-4159.2005.03522.x 16269007

[B68] van den Munckhof P. Luk K. C. Ste-Marie L. Montgomery J. Blanchet P. J. Sadikot A. F.. (2003). Pitx3 is required for motor activity and for survival of a subset of midbrain dopaminergic neurons. Development 130, 2535–2542. 10.1242/dev.00464 12702666

[B69] Wang Y. Zhou F. M. (2017). Striatal but not extrastriatal dopamine receptors are critical to dopaminergic motor stimulation. Front. Pharmacol. 8:935 10.3389/fphar.2017.00935 29311936 PMC5742616

[B70] Wei W. Ding S. Zhou F. M. (2017). Dopaminergic treatment weakens medium spiny neuron collateral inhibition in the parkinsonian striatum. J. Neurophysiol. 117, 987–999. 10.1152/jn.00683.2016 27927785 PMC5340884

[B71] Wei W. Li L. Yu G. Ding S. Li C. Zhou F. M. (2013). Supersensitive presynaptic dopamine D2 receptor inhibition of the striatopallidal projection in nigrostriatal dopamine-deficient mice. J. Neurophysiol. 110, 2203–2216. 10.1152/jn.00161.2013 23945778 PMC3841926

[B72] Xu H. Chen R. Cai X. He D. (2011). Differential effects of activating D1 and D2 receptors on electrophysiology of neostriatal neurons in a rat model of Parkinson's disease induced by paraquat and maneb. Neurosci Res. 71, 411–420. 10.1016/j.neures.2011.08.011 21903142

[B73] Yung K. K. Bolam J. P. Smith A. D. Hersch S. M. Ciliax B. J. Levey A. I. (1995). Immunocytochemical localization of D1 and D2 dopamine receptors in the basal ganglia of the rat: light and electron microscopy. Neuroscience 65, 709–730. 10.1016/0306-4522(94)00536-E 7609871

[B74] Zhou F. M (2017) The Substantia Nigra Pars Reticulata, in Handbook of Basal Ganglia Structure Function, 2nd Edn., eds Steiner H. Tseng K. (Amsterdam: Elsevier), 293–316.

[B75] Zhou F. M. Li L Yue J. Dani J. A. (2016). Transcription factor Pitx3 mutant mice as a model for Parkinson's disease. Front. Biol. 11:427. 10.1007/s11515-016-1429-826811110

[B76] Zhou Q. Y. Palmiter R. D. (1995). Dopamine-deficient mice are severely hypoactive, adipsic, and aphagic. Cell 83, 1197–1209. 10.1016/0092-8674(95)90145-0 8548806

